# Supplementary material for: The pharynx of the stem-chondrichthyan Ptomacanthus and the early evolution of the gnathostome gill skeleton
Source: Nat Commun. 2019 May 3;10:2050. doi: 10.1038/s41467-019-10032-3 (PMC6499890; doi:10.1038/s41467-019-10032-3)
Supplement: Supplementary file 1 — Supplementary Information [file 41467_2019_10032_MOESM1_ESM.pdf]

Supplementary Information

The pharynx of the stem-chondrichthyan *Ptomacanthus* and the early evolution of the gnathostome gill skeleton

Dearden et al.

This PDF file includes:

Supplementary Notes 1 and 2
Supplementary Figs. 1 to 5
Supplementary References

Supplementary Note 1: Supplementary text

The Mandibular Arch of *Ptomacanthus*

In addition to the branchial skeleton presented in the main text the specimen also preserves some information on the mandibular arch. Meckel's cartilages are visible overlapping slightly at the front of the specimen (Fig. 1b), although their exact shape is difficult to determine due to interference in the tomograms from the underlying gular squamation. There is no evidence for their ossification in two parts as described by Miles [1] supporting Brazeau's [2] suggestion that this effect is created in NHM specimen P 19999 by a stump of matrix. Both ventral tooth rows are preserved within the matrix, having become tipped and partially dissociated from the cartilage to varying degrees; these extend for at least half of the length of the mandible, diminishing in size posteriorly (Fig. 1b).

Further notes on the results of the parsimony analysis

The parsimony analysis we ran resulted in 78841 most parsimonious trees, with a length of 699 steps (Figs. 3, 4, Supplementary Fig. 4). The displacement of *Gladbachus* out of the acanthodian grade was supported unambiguously by the caudal extension of the pharynx, character 67, and by the relatively anterior extent of the pharynx, character 68, under ACCTRAN optimization, as well as unambiguously by the absence of a bony operculum (character 58).

Proposed reinterpretations of *Triodus sessilis*

Triodus sessilis is a Permian xenacanth in which the branchial arches are preserved. In recent analyses *Triodus* has been recovered as a relatively less-crownward stem-group elasmobranch [3]. The description of its branchial skeleton by Heidtke *et al.* [4] identified it as having had a broad basihyal and two anteriorly projecting hypohyals, in a manner not dissimilar to *Debeerius* [5].

On the basis of recently described chondrichthyan branchial skeletons, such as *Ptomacanthus*, and *Gladbachus*, we suggest that the proposed hypohyals of this skeleton could more parsimoniously interpreted as having instead been a U-shaped basihyal, similar to that in *Doliodus* [6], and to a lesser extent in *Gladbachus* [3]. The “basihyal” of Heidtke *et al.* could either be absent or comprise two hypohyals like those in *Gladbachus*. This interpretation would bring its branchial skeleton more in line with seems to be the plesiomorphic chondrichthyan condition from this and other studies, as well as removing the need to explain hypohyals (homoplasic or otherwise) in both the elasmobranch and the holocephalan crown-groups.

Pharyngeal patterning in early gnathostomes

In living jawed vertebrates a basihyal is only present in chondrichthyans (eg. Fig. 4i,k, *Scapanorhynchus* [7]). In early gnathostomes it is far more widespread. As well as being present in early crown-group and stem-group chondrichthyan branchial skeletons (eg. Fig. 4, *Gladbachus* [3,8], *Doliodus* [6], *Debeerius* [5] and in *Triodus* [4] a very similar element is also present in placoderm-grade stem-gnathostome branchial

skeletons (eg. *Paraplesiobatis* [9], *Cowralepis* [10], *Tapinosteus* [11]), and *Pseudopetalichthys* [9,12]. This phylogenetic distribution suggests that the presence of a basihyal is the ancestral state for gnathostomes, retained in chondrichthyans. The basihyal in *Ptomacanthus*, contacting the ceratohyals and first branchial arch, fits this pattern. It also adds credence to Gardiner's reconstruction of *Acanthodes* [13], with a similar arrangement contacting the hyoid and first branchial arch. From our observations of *Acanthodes* casts at the Natural History Museum, London, Gardiner's reconstruction seems to be the most credible. Early examples of osteichthyan branchial skeletons also often have a single median element - the "basibranchial" (eg. Fig. 4 *Raynerius* [14], *Glyptolepis* [15]). This is distinct from a basihyal in articulating with the hyoid arch via hypohyals, contacting more than one branchial arch, and extending further posteriorly in the pharynx [3]. However, it is possible that this and the basihyal are homologous.

In living osteichthyans an additional pair of bony struts - the hypohyals - connect the ceratohyals with the median basibranchial (Fig. 4). Hypohyals have long been identified as an unambiguous synapomorphy of osteichthyans [16,17] as they are entirely absent in extant chondrichthyans [7]. The report of hypohyal-like structures in various early chondrichthyans - particularly symmoriids [3,18–20], but also in the group more widely ([4,5]) has led to the suggestion that they are instead a gnathostome symplesiomorphy [20]. Evidence against their homology comes from the distinct anatomical differences in arrangement of osteichthyan and chondrichthyan hypohyals [21]. The absence of mineralised hypohyals in *Ptomacanthus* again corresponds with Gardiner's reconstruction of *Acanthodes*, as well as with their absence in *Gladbachus* [3]. This apparent absence of hypohyals on the chondrichthyan stem, their apparent absence in placoderms, and the topology recovered in our phylogenetic analysis suggest that osteichthyan hypohyals are apomorphic for the clade, and that chondrichthyan hypohyals are homoplasious, and apomorphic for early holocephalans.

A succession of paired and unpaired mineralisations support the floor of the pharynx posterior to the basihyal in living chondrichthyans (Fig. 4i,k). Paired elements - hypobranchials - are present in crown-chondrichthyans (and osteichthyans), with their posterior orientation in elasmobranchs possibly synapomorphic [3]. Meanwhile an unpaired basibranchial copula element is present in living holocephalans and elasmobranchs, as well as in fossil taxa including *Ozarcus*, *Gladbachus*, and *Triodus* [3,4,20], and could be apomorpic for a subset of total-group chondrichthyans. *Ptomacanthus* is distinct from these chondrichthyans in lacking any mineralised basi- or hypobranchial elements. Gardiner's reconstruction of *Acanthodes* also lacks posterior basibranchial elements, although it does possess paired hypobranchials on the posterior three branchial arches. Like *Ptomacanthus* and *Acanthodes*, "placoderm"-grade stem-gnathostomes also seemingly lack an unpaired mineralization posterior to the basihyal [9–11]. Instead a series of paired ventral elements with no space for median elements in between is present [9]. The homology of these elements is unclear - they are possibly homologous with median basibranchials [10] but in other taxa more closely resemble ceratobranchials [9]. While the homology of paired elements is poorly understood, a mineralized basibranchial copula is likely derived in chondrichthyans.

Other patterns comparable to other chondrichthyans can be observed in more dorsal parts of the gill skeleton of *Ptomacanthus* (Fig. 4). Despite the incompleteness of the

pharyngobranchials, they form an antero-posterior chain with dorsal ridges, like those in *Acanthodes*, *Ozarcus*, and *Gladbachus* [3,20]. Like living chondrichthyans, *Ptomacanthus* possesses five epibranchials [22]. The articulation of the posteriormost two of these on the same pharyngobranchial is very similar to the condition in *Ozarcus* [20], and is comparable to that in modern chondrichthyans where epibranchial V is fused to pharyngobranchial IV and articulates with epibranchial IV [22]. Finally the lack of any articulation between the two posteriormost ventral paired branchial arches in *Ptomacanthus* contrasts with a condition shared by *Paraplesiobatis* [9], and in various sarcopterygians such as *Glyptolepis* [15], which may be a gnathostome symplesiomorphy.

Supplementary Note 2: Phylogenetic dataset

Notes on taxa

For our phylogenetic analysis we added additional information to the dataset of Coates *et al.* [3] *Ptomacanthus* was scored for branchial characters on the basis of this work. Two additional taxon were added: *Paraplesiobatis*, a "placoderm" with three dimensionally preserved branchial arches [9], to increase sampling of stem-gnathostome branchial arches, and *Euthacanthus*, an acanthodian with a potentially important combination of climatiid-like and acanthodiform-like traits [23]. *Nereipisacanthus* was modified for a number of characters – notably character 225 from 0 to ?. *Latviacanthus* was changed from 1 to ? for character 228. *Ligulalepis* and *Ischnacanthus* had scoring updated on the basis of recent descriptions.

Sources used to score characters in addition to those listed by Coates *et al.* 2018 [3] are as follows:

Euthacanthus [23–25]
Ischnacanthus [26]
Ligulalepis [27]
Paraplesiobatis - [9,12], KGM 1983/294
Ptomacanthus – NHM P 24919a

Institutional abbreviations: **KGM** - Schlossparkmuseum (Karl-Geib-Museum), Bad Kreuznach, **NHM –** Natural History Museum, London

Notes on characters

We modified and added a number of characters from the original matrix, several of which relate to the pharyngeal skeleton: these are detailed below. Also included are recodings.

<u>MODIFIED Character 17</u> – Scale sensory line of body: (0) passes between or beneath scales, (1) passes over scales and/or is partially enclosed or surrounded by scales, (2) perforates or passes through scales

This replaces character 17 of Coates *et al.* "Sensory line canal…". The original character attempts to capture the different morphologies of in the sensory line canals in: (1) most chondrichthyans, where it passes between scales [28], (2) those of some early chondrichthyans (eg. *Akmonistion* [19]) which are carried by c-shaped scales, and of some acanthodians (eg. *Homalacanthus* [29]) where the head sensory lines are carried in open gutter-like scale, and (3) those of osteichthyans and stem-gnathostomes, where the canal passes through the scales themselves.

However, in doing so it conflates states in the head and body, which can differ in the same animal, particularly in acanthodians. In *Homalacanthus* for example the sensory lines of the head are carried by specialized scales, whereas lateral lines of the body pass between scales [29]. Here we have attempted to remedy this by distinguishing between two characters – one covering body squamation (17) and one for head squamation (18).

ADDED Character 18 - Scale sensory line of head: (0) passes between or beneath scales, (1) passes over scales and/or is partially enclosed or surrounded by scales, (2) perforates or passes through scales

See above.

MODIFIED Character 64 – Opercular flap/gill slits (0) complete operculum (1) separate gill covers and gill slits

Originally this character coded for the multiple separate gill slits on either side of the pharynx. In osteichthyans (and holocephalans) there is instead a single pair of gill openings, covered by an operculum. This is also the case in "placoderm" grade stem-gnathostomes. The previous formulation of this character considered it in terms of a partial/complete dermal covering vs separate uncovered slits. However, a dermal operculum is already accounted for with a different character, which records the presence or absence of bony hyoidean gill covers (character 58). Many "acanthodian" grade chondrichthyans (for example *Ptomacanthus*) have both a bony hyoidean gill cover, and multiple gill slits in the dermal integument [1,30], while living holocephalans have no dermal bony covering, but a single fleshy operculum [31]. For this reason we have explicitly linked this character to the number of openings to avoid redundancy. We have coded it conservatively - in many chondrichthyans, such as *Acanthodes* not enough of the dermal skeleton is judged to be preserved to be able to tell if multiple openings were present.

MODIFIED Character 67 Gill skeleton extends posteriorly beyond occiput (0) absent, (1) present

The branchial arches of osteichthyans and stem-gnathostomes are located beneath the neurocranium, whereas those of chondrichthyans have a pronounced posterior extension beyond the occiput (a notable, and certainly derived, exception being holocephalans). Coates *et al.* [3] capture this with their character number 66, present in conventional chondrichthyans and some acanthodians (eg. *Acanthodes*).

As discussed in the main text, here we consider that there is a subtlety to this character that is not considered – the anterior and posterior extents of the branchial skeleton. This is covered by this and the next character.

ADDED Character 68 First branchial arch meets neurocranium (1) ventral to otic region (1) posterior to otic region

See above

MODIFIED Character 73 - Anteriormost unpaired element of branchial skeleton contacted by: (0) one branchial arch only, (1) two or more branchial arches

This is modified from and replaces character 71 of Coates *et al.* [3]: Basihyal absent/present. A basihyal is a median unpaired component of the hyoid arch – in living gnathostomes it is only present in chondrichthyans, where it articulates with the hyoid arch, and sometimes the hyoid arch and first branchial arch [7,20]. This is

distinct from the basibranchial/s of osteichthyans, an unpaired element or group of elements onto which the hyoid arch (via hypohyals) and branchial arches articulate (eg. *Glyptolepis* [15], or *Raynerius* [14]). Amongst extinct gnathostomes a basihyal is usually considered present in chondrichthyans – both crown-group (eg. *Egertonodus* [32], and *Debeerius* [5]) and stem-group (eg. *Gladbachus* [3]) – as well as in various stem-gnathostomes (for example, *Paraplesiobatis* [9]).

The actual diagnosis for what constitutes a basihyal as opposed to a basibranchial in a fossil is slightly opaque - compare codings for Giles *et al.* [21] character 73 with Coates *et al.* [3] character 71. In their discussion of basihyals Coates et al. ([3] – supplementary material) state that the "position, connectedness, and separation from other basibranchials" are what distinguish a basihyal from a basibranchial/copula. This diagnosis is a compound combination of traits. Notwithstanding this the relative position of basihyals and basibranchials actually often differs little – the basibranchial of *Raynerius*, for example [14], is actually in much the same position relative to the hyoid arch as that of *Paraplesiobatis* [9] or *Ptomacanthus*.

Here we attempt to break the remainder of this diagnosis down into characters – this character and the following. One aspect of the connectedness of a "basihyal" is the absence of hypohyals – these are captured in the previous character. We also note that there are two additional characters one could try, but which are uninformative with the current dataset. The first could attempt to capture the spacing aspect of the basihyal definition *sensu* Coates *et al.* [3] a basihyal is usually well separated from the posterior basibranchial copulae in chondrichthyans – this is not observed in osteichthyans with posterior basibranchials (eg. *Amia* [33]), *Eusthenopteron*[34]).). The second could attempt to code for the articulation of only the hyoid arch onto the basihyal, a state which at least present in some holocephalan (pers. obvs).

This character distinguishes an attribute of connectedness that separates a basihyal *sensu* Coates *et al.* [3] from basibranchials/copulae – the number of branchial arches articulating with it in addition to the hyoid arch. In taxa with a "basihyal" this is either one or none, whereas in osteichthyans it is typically two or more.

<u>ADDED Character 74</u> – Multiple unpaired branchial mineralisations (0) absent, (1) present

Often gnathostomes have a chain of unpaired elements running along the base of the branchial basket – whether these be the copulae of chondrichthyans or the basibranchials of osteichthyans. *Ptomacanthus* lacks any kind of mineralised unpaired structure posterior to its basihyal. It shares this in common with placoderms such as *Paraplesiobatis* [9] and *Cowralepis* [10]. Contrastingly, in most chondrichthyans a basibranchial copula is present, eg. *Ozarcus* [20] and *Gladbachus* [3]. Osteichthyans vary between having a chain of elements (eg. *Amia* [33]) or a single element or complex of elements onto which all arches articulate (eg. *Mimipiscis* [13]or *Eusthenopteron* [34]). *Ozarcus* [20] has been scored 1 despite the apparent absence of a basihyal as its elongate posterior ossification seems likely to equate to the basibranchial copulae of other chondrichthyans.

<u>ADDED Character 80</u>: Posterior two ventral branchial arches (0) separate, (1) articulate ventrally

In the “placoderm” stem-gnathostome *Paraplesiobatis*, the posterior two branchial arches articulate ventrally [9]. A similar state is also observed in various sarcopterygians, such as *Glyptolepis* [15] and *Gryphognathus* [35]. Contrastingly, no such state is found in chondrichthyans, such as *Ozarcus* [20], *Gladbachus* [3], and now, *Ptomacanthus* . It is possible that having fully separated ventral paired branchial arches is autapomorphic for chondrichthyans.

ADDED Character 81: Posterior two dorsal branchial arches (0) separate, (1) articulate dorsally

In living chondrichthyans the posteriormost (fifth) epibranchial forms a fused complex with the fourth pharyngobranchial [22]. *Ptomacanthus* has a separate fifth epibranchial: however, rather than having its own expanded end (presumed pharyngobranchial) it articulates with the posterior of the fourth pharyngobranchial. A similar state is observed in the stem-holocephalan *Ozarcus* [20]. Thus this possibly constitutes an autapomorphy, at some level, of chondrichthyans.

MODIFIED Character 262: Anteriormost intermediate spine associated with shoulder girdle

This replaces Coates *et al.* [3] character 256: Admedian pectoral spines absent/present. Intermediate, or prepelvic, fin spines form a paired series between the pelvic and pectoral fins of many stem-chondrichthyans (eg. *Climatius, Doliodus*) [1,36,37]. In some animals a pair of spines is positioned anteriorly, between the pectoral fin spines (eg. *Vernicomacanthus* [1]). These were considered by Miles [1] to be the first pair of intermediate fin spines - more recently Burrow *et al.* [38] argued that they should instead be considered to be separate "admedian" fin spines. Given that these spines are in series with the intermediate spines where present and are seemingly not fixed to an endoskeletal element like other spines of the shoulder girdle we consider that they are most conservatively interpreted as being intermediate fin spines, and so have coded taxa with only these spines and no other intermediate spines as "present" for this character.

Although *Doliodus* is described by Maisey *et al.* [37] as having admedian spines, comparing its position on the scapulocoracoid to other "climatiids" [1] we consider their second suggestion, that it is a prepectoral spine, far more likely - it is scored accordingly.

Notes on analysis

To allow exact replication of our tnt analysis we have included a list of tnt commands used in the text file **PtomCommands.txt** in a .zip archive titled Supplementary_Software.zip. If a directory contains both the tnt matrix (**PtomMatrix.tnt**) and this text file (**PtomCommands.txt**) then these commands can be automatically run in tnt (downloadable here: http://www.lillo.org.ar/phylogeny/tnt/) by entering the following.

cdir INSERT DIRECTORY HERE;
proc PtomCommands.txt;

The commands used for MrBayes are included in a Bayes block in the NEXUS file **PtomBayes.nex**.

Supplementary Figure 1. **(a)** Dorsal view of the ventral branchial skeleton, with the groove in right ceratobranchial II shown (line runs approximately through groove) in **(b)** dorsal and **(c)** posterior aspect, **(d)** Dorsal view of the left hand dorsal branchial skeleton, with views of **(e)** the right hand anterior dorsal skeleton, **(f)** displaced fourth epibranchial, and **(g)** the junction between the fourth and fifth left epi/pharyngobranchials.

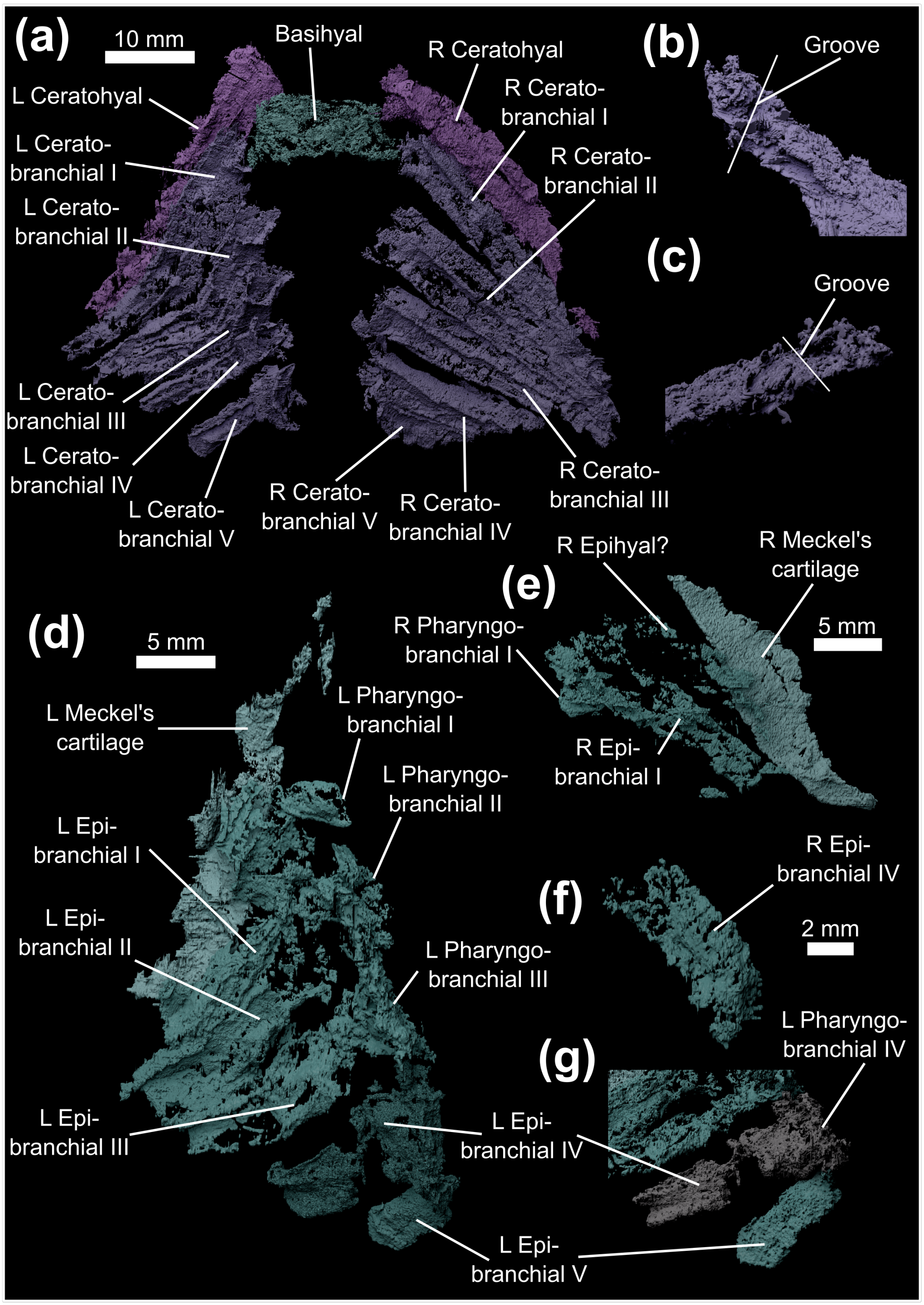

Supplementary Figure 2. Tomograms showing the basihyal in **(a)** the approximate sagittal plane, and reconstructed sections through **(b)** the approximate transverse plane, and **(c)** the approximate coronal plane. Abbreviations: **br. arch**, branchial arch; **cerhy.**, ceratohyal; **gul. squam.**, gular squamation; **Mck.**, Meckel's cartilage.

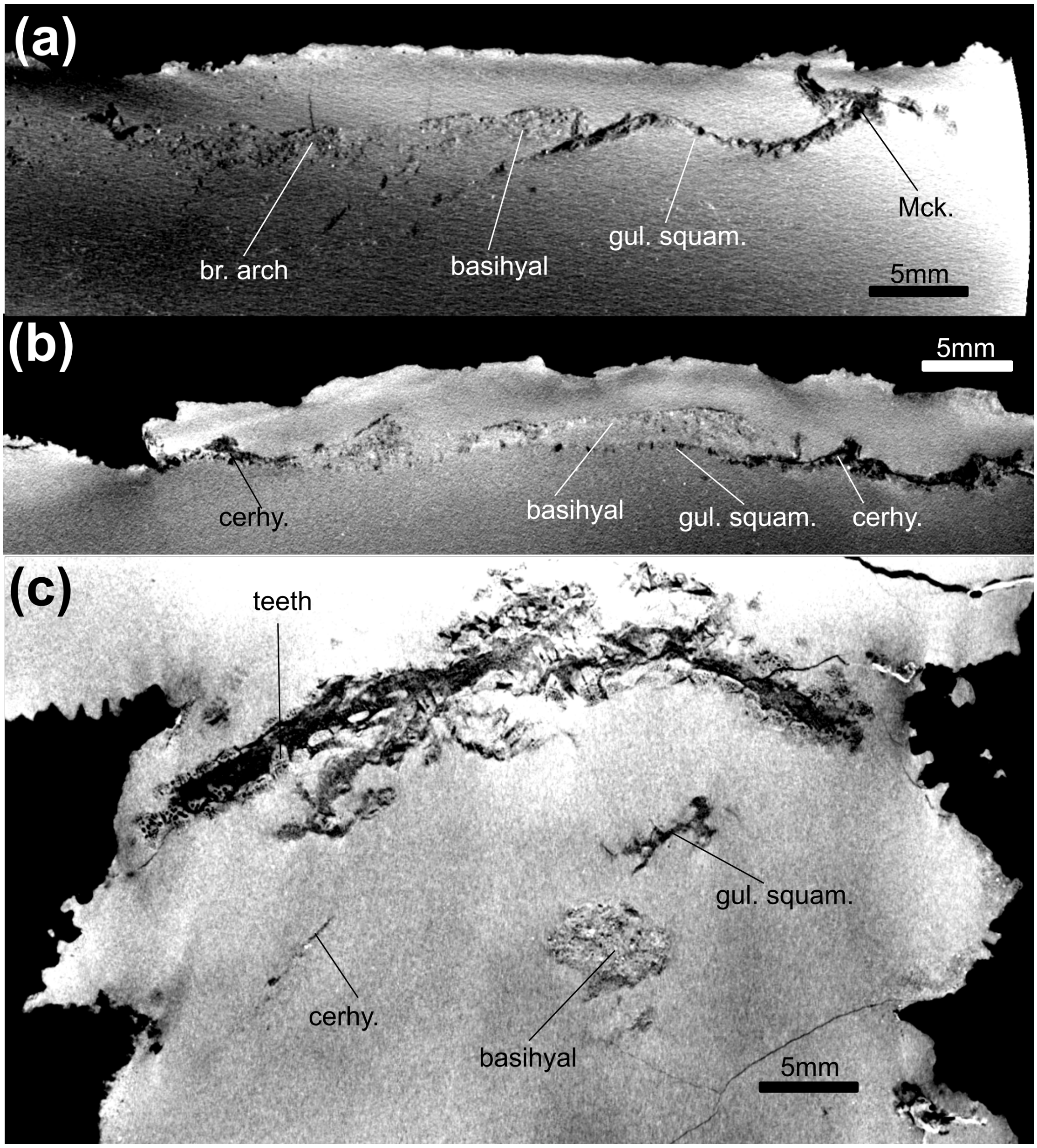

Supplementary Figure 3. Views of the surface mould of the specimen with **(a)** the entire branchial skeleton overlain, **(b)** the dorsal branchial skeleton overlain, and **(c)** no overlay. A video linking these views is also included in the supplementary material.

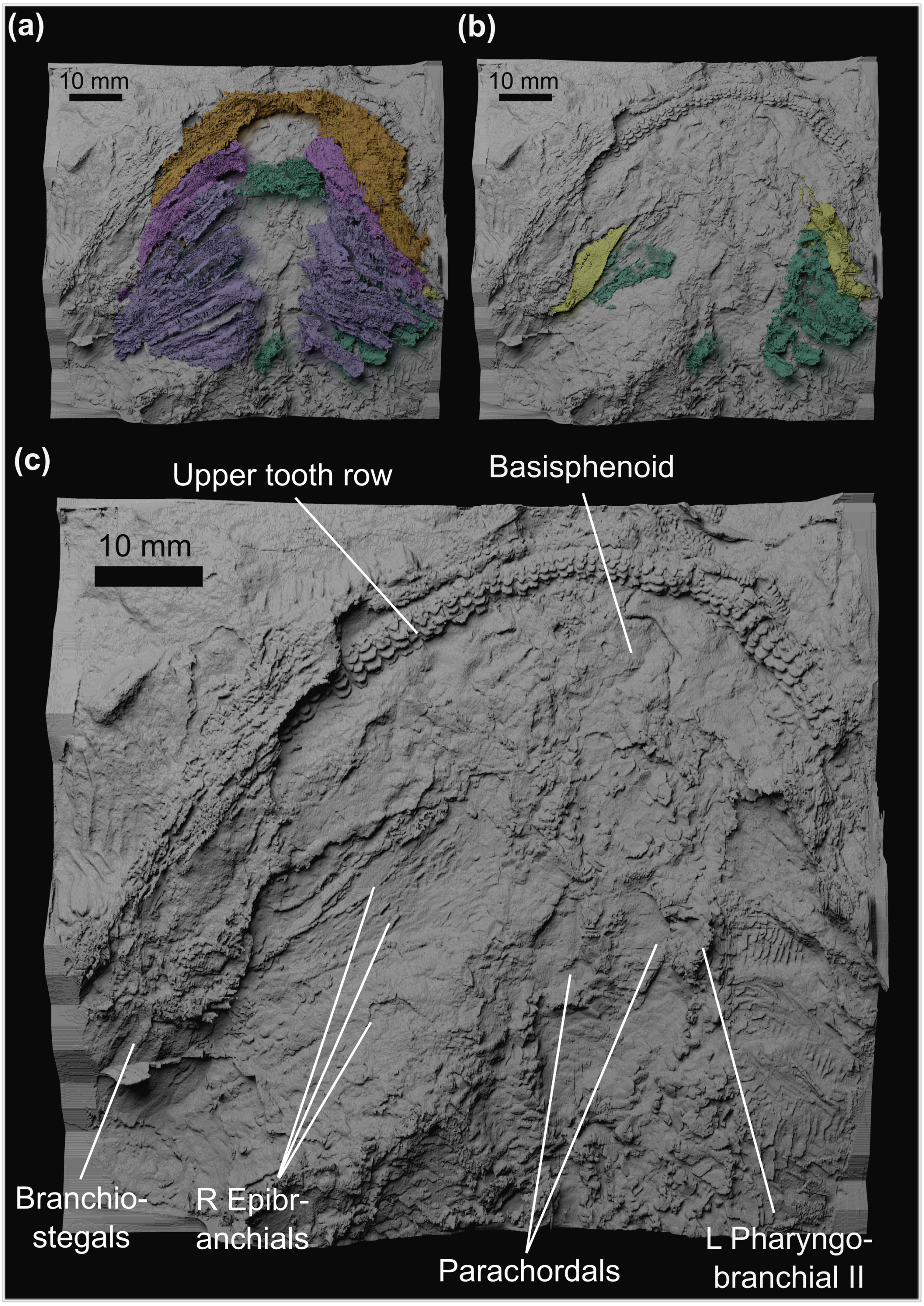

Supplementary Figure 4.(next page) The results of the parsimony analysis (a) the strict consensus tree, (b) the Adams consensus tree. The Adams tree includes nestings that are common to all trees, and so may contain groups that do not occur in any input tree [39]. Silhouettes from[3,13,30,40] and Phylopic. Phylopic images of *Bothriolepis* and *Callorhinchus milii* were submitted by Ghedoghedo and Tony Ayling (vectorised by Milton Tan) respectively, under a CC Attribution-ShareAlike 3.0 Unported license (https://creativecommons.org/licenses/by-sa/3.0/). Phylopic image of *Heterodontus* was submitted by M. Kolmann under a CC 1.0 Universal license (https://creativecommons.org/publicdomain/zero/1.0/).

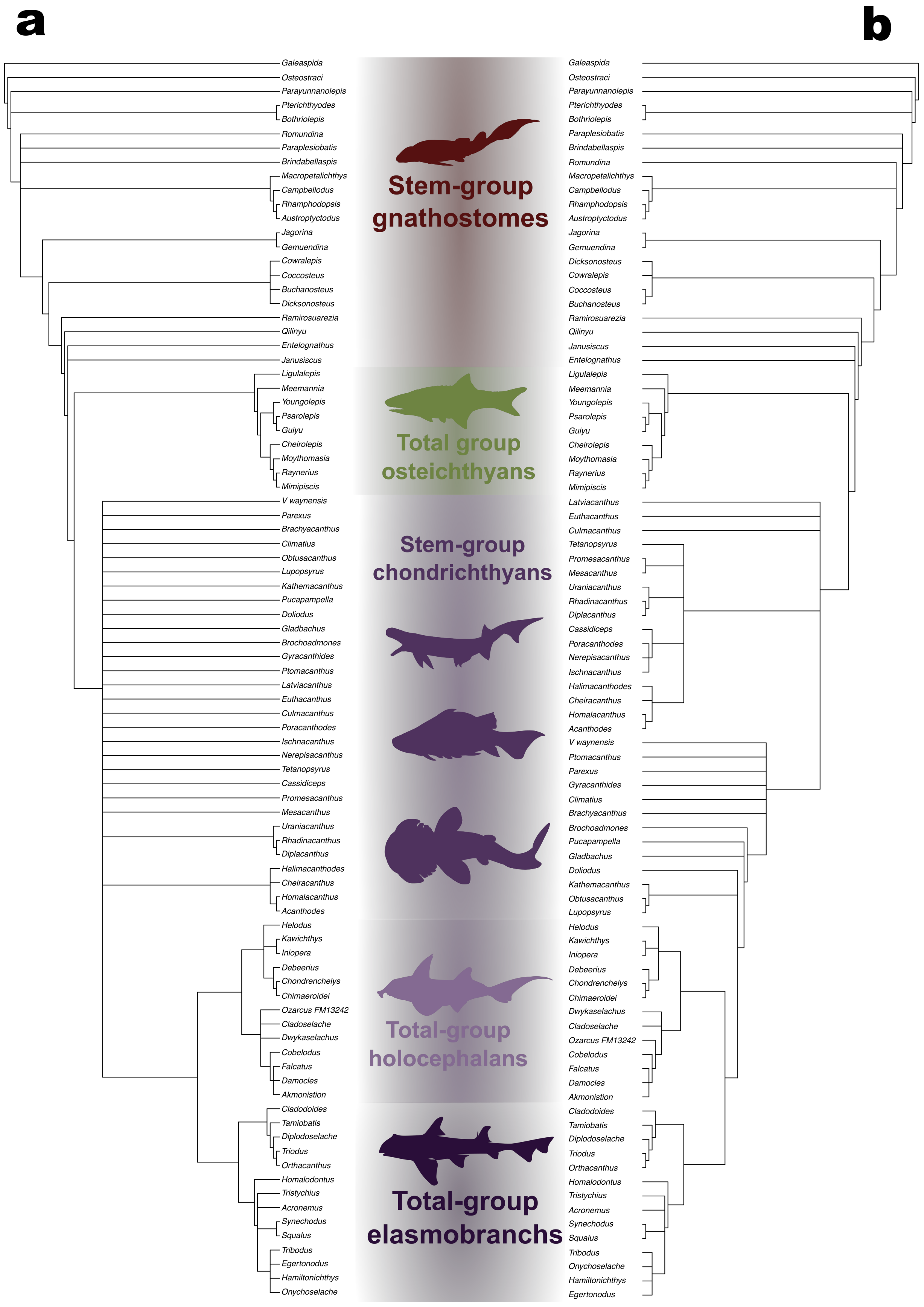
a
b
Stem-group gnathostomes
Total group osteichthyans
Stem-group chondrichthyans
Total-group holocephalans
Total-group elasmobranchs
Galeaspida
Osteostraci
Parayunnanolepis
Pterichthyodes
Bothriolepis
Romundina
Paraplesiobatis
Brindabellaspis
Macropetalichthys
Campbellodus
Rhamphodopsis
Austroptyctodus
Jagorina
Gemuendina
Cowralepis
Coccosteus
Buchanosteus
Dicksonosteus
Ramirosuarezia
Qilinyu
Entelognathus
Janusiscus
Ligulalepis
Meemannia
Youngolepis
Psarolepis
Guiyu
Cheirolepis
Moythomasia
Raynerius
Mimipiscis
V waynensis
Parexus
Brachyacanthus
Climatius
Obtusacanthus
Lupopsyrus
Kathemacanthus
Pucapampella
Doliodus
Gladbachus
Brochoadmones
Gyracanthides
Ptomacanthus
Latviacanthus
Euthacanthus
Culmacanthus
Poracanthodes
Ischnacanthus
Nerepisacanthus
Tetanopsyrus
Cassidiceps
Promesacanthus
Mesacanthus
Uraniacanthus
Rhadinacanthus
Diplacanthus
Halimacanthodes
Cheiracanthus
Homalacanthus
Acanthodes
Helodus
Kawichthys
Iniopera
Debeerius
Chondrenchelys
Chimaeroidei
Ozarcus FM13242
Cladoselache
Dwykaselachus
Cobelodus
Falcatus
Damocles
Akmonistion
Cladododoides
Tamiobatis
Diplodoselache
Triodus
Orthacanthus
Homalodontus
Tristychius
Acronemus
Synechodus
Squalus
Tribodus
Egertonodus
Hamiltonichthys
Onychoselache
Galeaspida
Osteostraci
Parayunnanolepis
Pterichthyodes
Bothriolepis
Paraplesiobatis
Brindabellaspis
Romundina
Macropetalichthys
Campbellodus
Rhamphodopsis
Austroptyctodus
Jagorina
Gemuendina
Dicksonosteus
Cowralepis
Coccosteus
Buchanosteus
Ramirosuarezia
Qilinyu
Janusiscus
Entelognathus
Ligulalepis
Meemannia
Youngolepis
Psarolepis
Guiyu
Cheirolepis
Moythomasia
Raynerius
Mimipiscis
Latviacanthus
Euthacanthus
Culmacanthus
Tetanopsyrus
Promesacanthus
Mesacanthus
Uraniacanthus
Rhadinacanthus
Diplacanthus
Cassidiceps
Poracanthodes
Nerepisacanthus
Ischnacanthus
Halimacanthodes
Cheiracanthus
Homalacanthus
Acanthodes
V waynensis
Ptomacanthus
Parexus
Gyracanthides
Climatius
Brachyacanthus
Brochoadmones
Pucapampella
Gladbachus
Doliodus
Kathemacanthus
Obtusacanthus
Lupopsyrus
Helodus
Kawichthys
Iniopera
Debeerius
Chondrenchelys
Chimaeroidei
Dwykaselachus
Cladoselache
Ozarcus FM13242
Cobelodus
Falcatus
Damocles
Akmonistion
Cladododoides
Tamiobatis
Diplodoselache
Triodus
Orthacanthus
Homalodontus
Tristychius
Acronemus
Synechodus
Squalus
Tribodus
Onychoselache
Hamiltonichthys
Egertonodus

Supplementary Figure 5. The majority rule consensus tree of the Bayesian analysis, with nodes labelled with posterior probabilities - the percentage of sampled trees recovering that split. Silhouettes from[3,13,30,40] and Phylopic. Phylopic images of *Bothriolepis* and *Callorhinchus milii* were submitted by Ghedoghedo and Tony Ayling (vectorised by Milton Tan) respectively, under a CCAttribution-ShareAlike 3.0 Unported license (https://creativecommons.org/licenses/by-sa/3.0/). Phylopic image of *Heterodontus* was submitted by M. Kolmann under a CC 1.0 Universal license (https://creativecommons.org/publicdomain/zero/1.0/).

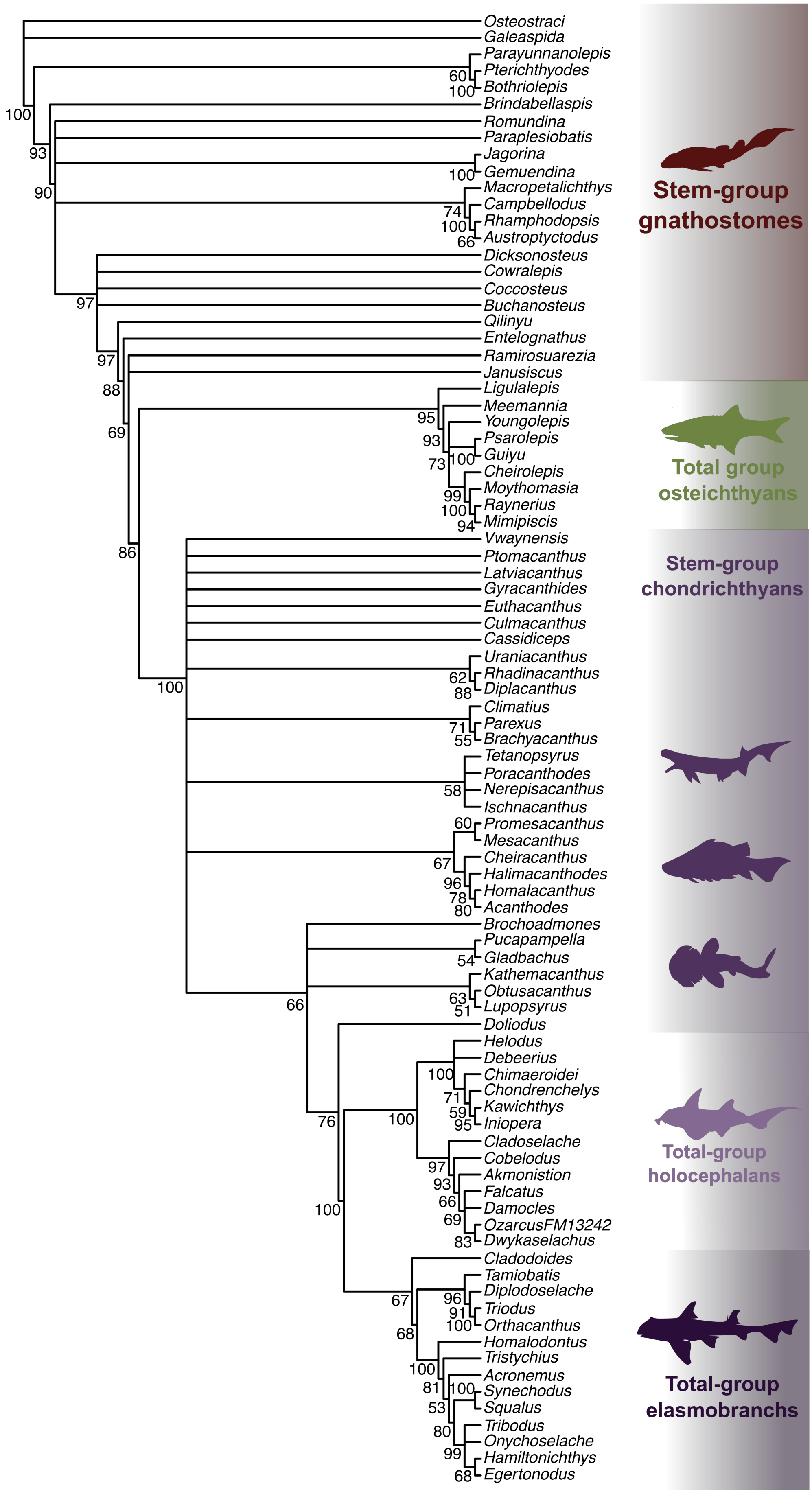
Osteostraci
Galeaspida
Parayunnanolepis
Pterichthyodes
Bothriolepis
Brindabellaspis
Romundina
Paraplesiobatis
Jagorina
Gemuendina
Macropetalichthys
Campbellodus
Rhamphodopsis
Austroptyctodus
Dicksonosteus
Cowralepis
Coccosteus
Buchanosteus
Qilinyu
Entelognathus
Ramirosuarezia
Janusiscus
Ligulalepis
Meemannia
Youngolepis
Psarolepis
Guiyu
Cheirolepis
Moythomasia
Raynerius
Mimipiscis
Vwaynensis
Ptomacanthus
Latviacanthus
Gyracanthides
Euthacanthus
Culmacanthus
Cassidiceps
Uraniacanthus
Rhadinacanthus
Diplacanthus
Climatius
Parexus
Brachyacanthus
Tetanopsyrus
Poracanthodes
Nerepisacanthus
Ischnacanthus
Promesacanthus
Mesacanthus
Cheiracanthus
Halimacanthodes
Homalacanthus
Acanthodes
Brochoadmones
Pucapampella
Gladbachus
Kathemacanthus
Obtusacanthus
Lupopsyrus
Doliodus
Helodus
Debeerius
Chimaeroidei
Chondrenchelys
Kawichthys
Iniopera
Cladoselache
Cobelodus
Akmonistion
Falcatus
Damocles
OzarcusFM13242
Dwykaselachus
Cladodoides
Tamiobatis
Diplodoselache
Triodus
Orthacanthus
Homalodontus
Tristychius
Acronemus
Synechodus
Squalus
Tribodus
Onychoselache
Hamiltonichthys
Egertonodus
Stem-group gnathostomes
Total group osteichthyans
Stem-group chondrichthyans
Total-group holocephalans
Total-group elasmobranchs

Supplementary References

1. Miles, R. S. Articulated acanthodian fishes from the Old Red Sandstone of England, with a review of the structure and evolution of the acanthodian shoulder girdle. *Bull. Br. Museum (Natural Hist. Geol.* **24,** (1973).
2. Brazeau, M. D. The braincase and jaws of a Devonian 'acanthodian' and modern gnathostome origins. *Nature* **457,** 305–308 (2009).
3. Coates, M. I. *et al.* An early chondrichthyan and the evolutionary assembly of a shark body plan. *Proc. R. Soc. B Biol. Sci.* **285,** (2018).
4. Heidtke, U. H. J., Schwind, C. & Krätschmer, K. Über die Organisation des Skelettes und die verwandschaftlichen Beziehungen der Gattung *Triodus* JORDAN 1849 (Elasmobranchii: Xenacanthida). *Mainzer Geowissenschaftliche Mitteilungen* **32,** 9–54 (2004).
5. Grogan, E. D. & Lund, R. *Debeerius ellefseni* (fam. nov., gen. nov., spec. nov.), an autodiastylic chondrichthyan from the Mississippian Bear Gulch Limestone of Montana (USA), the relationships of the Chondrichthyes, and comments on gnathostome evolution. *J. Morphol.* **245,** 219–245 (2000).
6. Miller, R. F., Cloutier, R. & Turner, S. The oldest articulated chondrichthyan from the Early Devonian period. *Nature* **425,** 501–504 (2003).
7. Garman, S. The Plagiostomia: Sharks, skates and rays. *Mem. Museum Comp. Zool. Harvard Coll.* **36,** 1–528 (1913).
8. Heidtke, U. H. J. & Krätschmer, K. *Gladbachus adentatus* nov. gen. et sp., ein primitiver Hai aus dem Oberen Givetium (Oberes Mitteldevon) der Bergisch Gladbach - Paffrath-Mulde (Rheinisches Schiefergebirge). *Mainzer Geowissenschaftliche Mitteilungen* **30,** 105–122 (2001).
9. Brazeau, M. D., Friedman, M., Jerve, A. & Atwood, R. A three-dimensional placoderm (stem-group gnathostome) pharyngeal skeleton and its implications for primitive gnathostome pharyngeal architecture. *J. Morphol.* **278,** 1–9 (2017).
10. Carr, R., Johanson, Z. & Ritchie, A. The phyllolepid placoderm *Cowralepis mclachlani*: insights into the evolution of feeding mechanisms in jawed vertebrates. *J. Morphol.* **270,** 775–804 (2009).
11. Stensiö, E. in *Traité de paleontologie* (ed. Piveteau, J.) **4,** 71–692 (Masson et Cie., 1969).
12. Gross, W. Neuuntersuchung der Stensiöellida (Arthrodira, Unterdevon). *Notizblatt des Hess. Landesamtes für Bodenforsch. zu Wiesbad.* **90,** 48–86 (1962).
13. Gardiner, B. G. The relationships of the palaeoniscid fishes, a review based on new specimens of *Mimia* and *Moythomasia* from the Upper Devonian of Western Australia. *Bull. Br. Museum (Natural Hist. Geol.* **37,** 175–418 (1984).
14. Giles, S., Darras, L., Clément, G., Blieck, A. R. M. & Friedman, M. An exceptionally preserved Late Devonian actinopterygian provides a new model for primitive cranial anatomy in ray-finned fishes. *Proc. R. Soc. B Biol. Sci.* **282,** (2015).
15. Jarvik, E. Middle and Upper Devonian Porolepiformes from East Greenland with special reference to *Glyptolepis groenlandica* n. sp. *Meddr. Grønl.* **187,** (1972).
16. Schaeffer, B. in *Current problems of lower vertebrate phylogeny. Proc. Fourth Nobel Symp.* (ed. Ørvig, T.) 207–222 (Armqvist and Wiksell, 1968).
17. Friedman, M. & Brazeau, M. D. A reappraisal of the origin and basal radiation of the Osteichthyes. *J. Vertebr. Paleontol.* **30,** 36–56 (2010).
18. Zangerl, R. & Case, G. Cobelodus aculeatus (Cope), an anacanthous shark from Pennsylvanian black shales of North America. *Palaeontogr. Abteilung A,*

Palaeozoologie-Stratigraphie **154,** 107–157 (1976).
19. Coates, M. I. & Sequeira, S. E. K. A new stethacanthid chondrichthyan from the lower Carboniferous of Bearsden , Scotland. *J. Vertebr. Paleontol.* 37–41 (2001). doi:10.1671/0272-4634(2001)021
20. Pradel, A., Maisey, J. G., Tafforeau, P., Mapes, R. H. & Mallatt, J. A Palaeozoic shark with osteichthyan-like branchial arches. *Nature* **509,** 608–11 (2014).
21. Giles, S., Friedman, M. & Brazeau, M. D. Osteichthyan-like cranial conditions in an Early Devonian stem gnathostome. *Nature* **520,** 82–175 (2015).
22. Carvalho, M., Bockmann, F. A. & de Carvalho, M. R. Homology of the fifth epibranchial and accessory elements of the ceratobranchials among Gnathostomes: Insights from the development of ostariophysans. *PLoS One* **8,** (2013).
23. Watson, D. M. S. The Acanthodian Fishes. *Philos. Trans. R. Soc. Lond. B. Biol. Sci.* **228,** 49–146 (1937).
24. Newman, M. J., Davidson, R. G., Den Blaauwen, J. L. & Burrow, C. J. The Early Devonian Acanthodian *Euthacanthus gracilis* from the Midland Valley of Scotland. *Scottish J. Geol.* **47,** 101–111 (2011).
25. Newman, M. J., Burrow, C. J., Den Blaauwen, J. L. & Davidson, R. G. The Early Devonian Acanthodian *Euthacanthus macnicoli* from the Midland Valley of Scotland. *Geodiversitas* **36,** 321–348 (2014).
26. Burrow, C. J., Newman, M., den Blaauwen, J., Jones, R. & Davidson, R. G. The Early Devonian ischnacanthiform acanthodian *Ischnacanthus gracilis* (Egerton, 1861) from the Midland Valley of Scotland. *Acta Geol. Pol.* **68,** 335–362 (2018).
27. Clement, A. M. *et al.* Neurocranial anatomy of an enigmatic Early Devonian fish sheds light on early osteichthyan evolution. *Elife* 1–28 (2018). doi:https://doi.org/10.7554/eLife.34349.001
28. Brazeau, M. D. & Friedman, M. The characters of Palaeozoic jawed vertebrates. *Zool. J. Linn. Soc.* **170,** 779–821 (2014).
29. Gagnier, P.-Y. in *Devonian fishes and plants of Miguasha, Quebec, Canada* 149–164 (1996).
30. Brazeau, M. D. A revision of the anatomy of the Early Devonian jawed vertebrate *Ptomacanthus anglicus* Miles. *Palaeontology* **55,** 355–367 (2012).
31. Didier, D. A. Phylogenetic Systematics of Extant Chimaeroid Fishes (Holocephali, Chimaeroidei). *Am. Museum Novit.* (1995).
32. Maisey, J. G. Cranial anatomy of Hybodus basanus Egerton from the Lower Cretaceous of England. *Am. Museum Novit.* 1–64 (1983).
33. Allis, E. P. The cranial muscles of *Amia*. *J. Morphol.* **XII,** (1897).
34. Jarvik, E. *Basic structure and evolution of vertebrates.* (Academic Press, 1980).
35. Miles, R. S. Dipnoan (lungfish) skulls and the relationships of the group : a study based on new species from the Devonian of Australia. *Zool. J. Linn. Soc.* **61,** 1–328 (1977).
36. Burrow, C. J., Davidson, R. G., Den Blaauwen, J. L. & Newman, M. J. Revision of *Climatius reticulatus* Agassiz, 1844 (Acanthodii, Climatiidae), from the Lower Devonian of Scotland, based on new histological and morphological data. *J. Vertebr. Paleontol.* **35,** e913421 (2015).
37. Maisey, J. G., Miller, R. F., Pradel, A., Denton, J. S. S. & Janvier, P. Pectoral Morphology in *Doliodus* : Bridging the 'Acanthodian'- Chondrichthyan Divide. *Am. Museum Novit.* 1–15 (2017).
38. Burrow, C. J., Blaauwen, J. Den, Newman, M. J. & Davidson, R. G. The diplacanthid

fishes (Acanthodii , Diplacanthiformes , Diplacanthidae) from the Middle Devonian of Scotland. *Palaeontol. Electron.* 1–83 (2016).
39. Wilkinson, M. Three-taxon statements: when is parsimony analysis also a clique analysis? *Cladistics* **10,** 221–223 (1994).
40. Long, J. A. A new Late Devonian acanthodian fish from Mt. Howitt, Victoria, Australia, with remarks on acanthodian biogeography. *Proc. R. Soc. Victoria* **98,** 1–17 (1986).
