# Supplementary material for: The pharynx of the stem-chondrichthyan Ptomacanthus and the early evolution of the gnathostome gill skeleton
Source: Nat Commun. 2019 May 3;10:2050. doi: 10.1038/s41467-019-10032-3 (PMC6499890; doi:10.1038/s41467-019-10032-3)
Supplement: Supplementary file 3 — Description of Additional Supplementary Files [file 41467_2019_10032_MOESM3_ESM.pdf]

Description of Additional Supplementary Files

File Name: Supplementary Movie 1
Description: Rendered ct model of the pharyngeal skeleton of *Ptomacanthus anglicus* viewed ventrally, against the mould of the surface of the specimen.

File Name: Supplementary Movie 2
Description: Rendered ct model of the pharyngeal skeleton of *Ptomacanthus anglicus* in isolate, rotating around its longitudinal axis.

File Name: Supplementary Software 1
Description: Data matrices and phylogenetic analysis command scripts.
